# Assessing the Correlation Between the Lumbar Disc Herniation Degree and Multifidus Muscle Fatty Degeneration in Chronic Low Back Pain

**DOI:** 10.7759/cureus.71237

**Published:** 2024-10-10

**Authors:** Elif Balevi Batur

**Affiliations:** 1 Physical Medicine and Rehabilitation, Selcuk University School of Medicine, Konya, TUR

**Keywords:** chronic pain, disc herniation, fatty degeneration, low back pain, magnetic resonance imaging, multifidus

## Abstract

Background: Chronic low back pain (LBP) is a common condition primarily associated with lumbar disc herniation. Fatty degeneration of the multifidus muscle is also observed in the majority (>80%) of patients with LBP.

Methods: This retrospective study included 140 patients (72 females and 68 males) with chronic LBP. 3T lumbar magnetic resonance imaging (MRI) T1- and T2-weighted fast spin-echo sequences were used for radiological evaluation. Disc herniations were graded according to the degree of bulging, protrusion, extrusion, and sequestration. Multifidus fatty infiltration was semiquantitatively graded from grade 1 (normal) to grade 4 (severe).

Results: Multifidus fatty degeneration was more common in females compared to males at the L4-L5 and L5-S1 levels (p-values <000.1 and <000.1, respectively). There was no correlation between disc herniation at the L4-L5 and L5-S1 levels and fatty degeneration of the multifidus muscle (p values 0.426 and 0.170). There was a statistically significant positive correlation between multifidus fatty degeneration at L4-L5 and L5-S1 levels and increasing age (p<000.1, r-values 0.462 and 0.357, respectively). In contrast, there was no correlation between age and L4-L5 and L5-S1 herniation grades (p values 0.167 and 0.723, respectively). Fatty degeneration grades were generally higher in females and increased with age at L4-L5 and L5-S1 levels in both genders (p<0.001).

Conclusions: The frequency and degree of fatty degeneration of the multifidus muscle increase with age, especially in women, and do not correlate with the presence of a herniated disc.

## Introduction

Lumbar disc herniation (LDH) plays a major role in causing low back pain (LBP) and sciatica in adults, typically affecting the L4/5 and L5/S1 lumbar segments and leading to symptoms such as radicular pain, sensory deficits, and muscle weakness [1]. Lumbar stability and degeneration are among the extensively researched factors that contribute to LDH. The multifidus muscle in the paravertebral group is a key player in distributing forces across intervertebral discs, ultimately boosting spinal stability during loading and compression [2]. The multifidus muscle is the first to be activated in instances of spinal instability, leading to heightened tension in the lumbar segments and minimizing excessive displacement and rotation of the spinal segments.

Various studies have explored the atrophy and fatty infiltration of the lumbar multifidus muscle (LMM) related to low back injury. A cross-sectional MRI-based study involving 412 adults and 442 adolescents identified patients with LBP and categorized LMM atrophy as none, slight, or severe. The study concluded a robust association between LMM fat infiltration and adult LBP [3]. Additionally, a retrospective study by Kader et al., involving 78 LBP patients, with or without leg pain, found that 80% of patients with LBP exhibited LMM atrophy detected by MRI, which correlated with leg pain [4]. MRI can identify these fatty atrophic changes in muscle, presenting as high-intensity regions located medially and deeply within the myofascial sheath of the LMM [3].

Intervertebral disc herniation is primarily associated with LBP and often results in spinal nerve root compression, leading to denervation, followed by atrophy and fatty infiltration of the multifidus muscle. Moreover, changes in paraspinal histological fibrosis patterns have been observed in disc herniation patients. Furthermore, avoiding activities that exacerbate pain stemming from herniation may induce disuse-related changes that further deteriorate these degenerative characteristics [5-7].

Recent studies have suggested a potential relationship between LDH and multifidus muscle degeneration in the lumbar region, including lower lumbar segments [8,9]. However, a previous study highlighted a positive correlation between disc degeneration and LMM atrophy, specifically at the L3-L4 disc level, but no correlation was detected at the lower lumbar segments [5]. Regarding the conflicting findings in the literature, this study aims to investigate the relationship between disc degeneration and fatty degeneration of the multifidus muscle at the L4-5 and L5-S1 levels, where lumbar disc herniation is most prevalent.

## Materials and methods

A retrospective analysis was conducted on 140 eligible patients (72 females and 68 males) aged between 20 and 75 years, all suffering from chronic LBP, defined as back pain persisting for over three months. Exclusion criteria included spinal fractures, prior spinal surgery, spinal cord injuries, spinal infections, spinal tumors, vertebral deformities such as kyphosis and scoliosis, and rheumatic diseases involving the spine. This study was conducted at and approved by the local ethics committee of Selcuk University Faculty of Medicine (approval number 2024/288). The lumbar MRI images of the patients who visited the outpatient clinic with chronic LBP were retrospectively analyzed between April and June 2024.

Imaging scans were performed using a 3-Tesla MRI machine (Magnetom Skyra, Siemens Healthineers AG, Munich, Germany). Evaluation of LMM fatty degeneration focused on axial sections at the L4-L5 and L5-S1 levels, as these levels typically exhibit the largest diameter of the LMM. T1 and T2 weighted fast spin-echo sequences were utilized, including T2 TSE sagittal, T1 TSE sagittal, and T2 TSE axial images.

**Figure 1:**
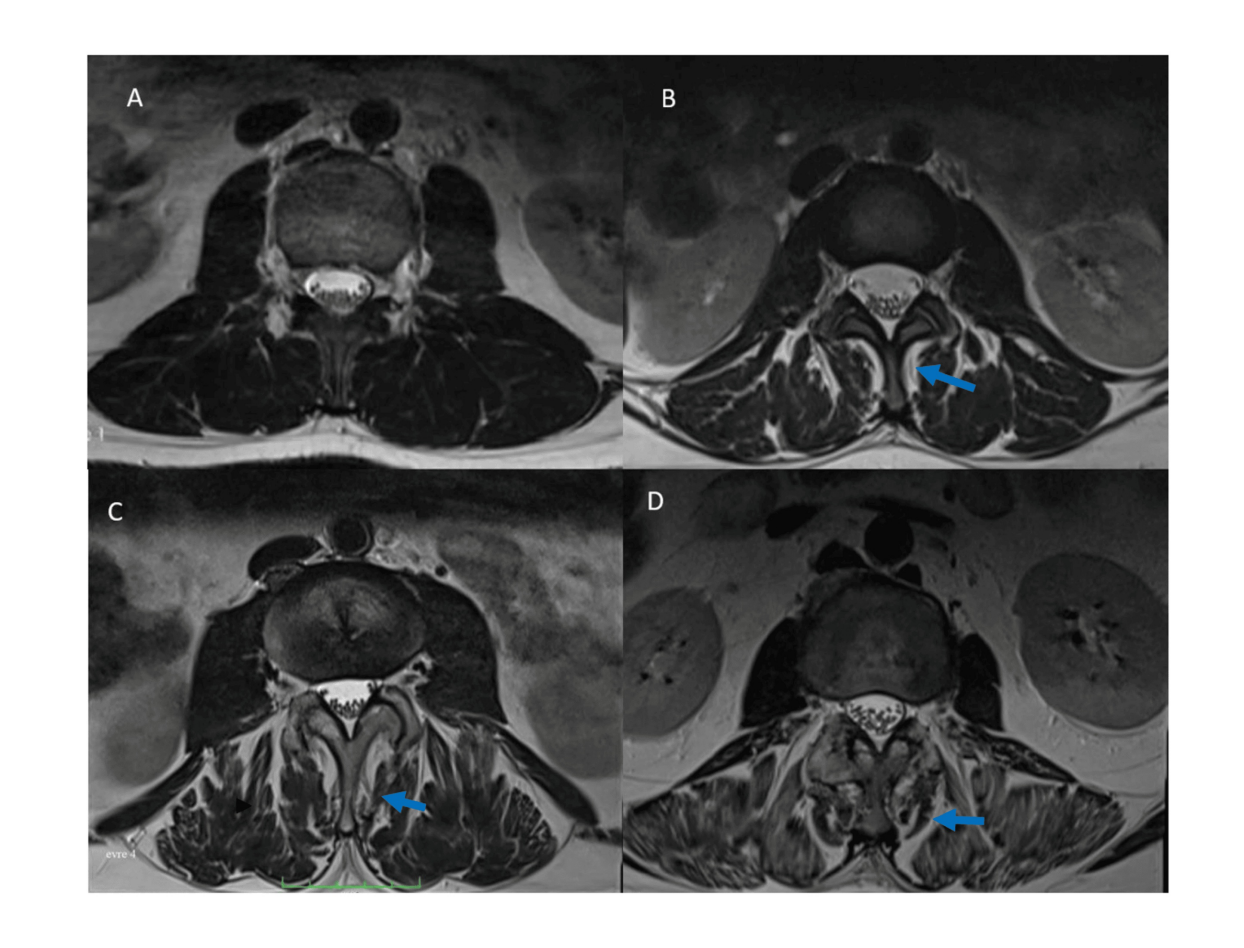
Fatty degeneration grades of the lumbar multifidus muscle A. Normal, B. Mild, C. Moderate, and D. Severe *Fatty degeneration areas are highlighted with an arrow in each image

Fatty degeneration of the LMM was assessed using T2-weighted axial MRI scans and categorized into four grades based on the extent of fatty infiltration: Grade 1 indicating normal muscle with up to 10% fatty infiltration, Grade 2 representing mild muscle degeneration with 10-30% fatty infiltration, Grade 3 indicating moderate degeneration with 30-50% fat infiltration, and Grade 4 signifying severe muscle atrophy with over 50% fatty infiltration, as described in the study by Faur et al. (Figure 1) [10].

The disc herniation type and location were evaluated in both axial and sagittal images. Disc herniation was classified based on the degree of bulging, protrusion, extrusion, and sequestration.

Statistical analysis

All statistical analyses were conducted using IBM SPSS Statistics for Windows, Version 22 (Released 2017; IBM Corp., Armonk, New York, United States). Parametric data are presented as mean ± standard deviation, while nonparametric data are expressed as median (minimum to maximum, min-max). Categorical variables are reported as numbers and percentages. The normality of the variables was assessed using the Kolmogorov-Smirnov test and Q-Q plots. Differences in parameters between groups were evaluated using the chi-square test or Fisher's exact test. Spearman's rank correlation test was used to assess associations between age, gender, herniation, and atrophy levels. Post hoc comparisons following ANOVA were conducted using the Hochberg test. A significance level of p < 0.05 was considered statistically significant.

## Results

The patient population had an average age of 47 years. There was no significant difference in the degree of herniation between males and females, as shown in Table 1.

**Table 1:** Demographic features and disc herniation degrees at L4-5 and L5-S1 levels in both genders ^a^chi-square test, ^b^Fisher’s exact test

Feature		Total: 140 patients	Female 72 (51.4%)	Male 68 (48.6%)	p-value
Age (Min-Max)		47 (20-80)	47 (23-80)	44.5 (20-77)	-
Herniation levels					
L4-L5	Normal	14 (10%)	8 (11.1%)	6 (8.8%)	0.116^a^
	Bulging	86 (61.4%)	48 (66.7%)	38 (55.9%)	
	Protrusion	26 (18.6%)	13 (18.1%)	13 (19.1%)	
	Extrusion	14 (10%)	3 (4.2%)	11 (16.2%)	
L5-S1	Normal	32 (22.9%)	21 (29.2%)	11 (16.2%)	0 .192^b^
	Bulging	71 (50.7%)	35 (48.6%)	36 (52.9%)	
	Protrusion	30 (21.4%)	11 (15.3%)	19 (27.9%)	
	Extrusion	7 (5%)	5 (6.9%)	2 (2.9%)	

However, females were found to have more advanced fatty degeneration in their low back muscles at the L4-L5 and L5-S1 levels compared to males (p values <000.1, <000.1, respectively) (Table 2).

**Table 2:** Distribution of lumbar multifidus muscle fatty degeneration grades at L4-L5 and L5-S1 levels in females and males *Pearson chi-square. Data are presented as n (%)

	Female, n=72 (51.4%)			Male, n=68 (48.6%)			
Fatty degeneration grade	Normal	Mild	Moderate + Severe	Normal	Mild	Moderate + Severe	p
L4-L5 level	9	43	20	25	38	5	<0.001*
L5-S1 level	1	43	28	14	42	12	<0.001*

**Figure 2:**
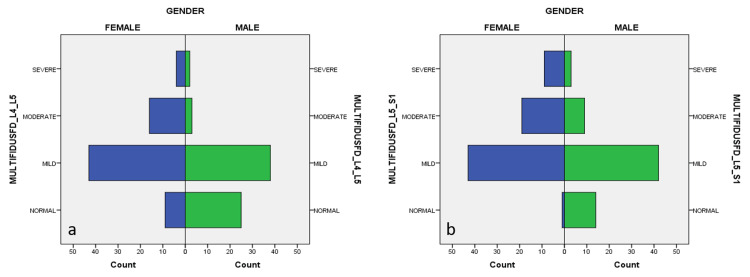
Gender and lumbar multifidus muscle fatty degeneration (FD) at L4-5 and L5-S1 levels a. L4-5 level, b. L5-S1 level

Additionally, multifidus muscle fatty degeneration was more common in females than males at both levels (p-values <000.1 and <000.1, respectively) (Figure 2).

**Figure 3:**
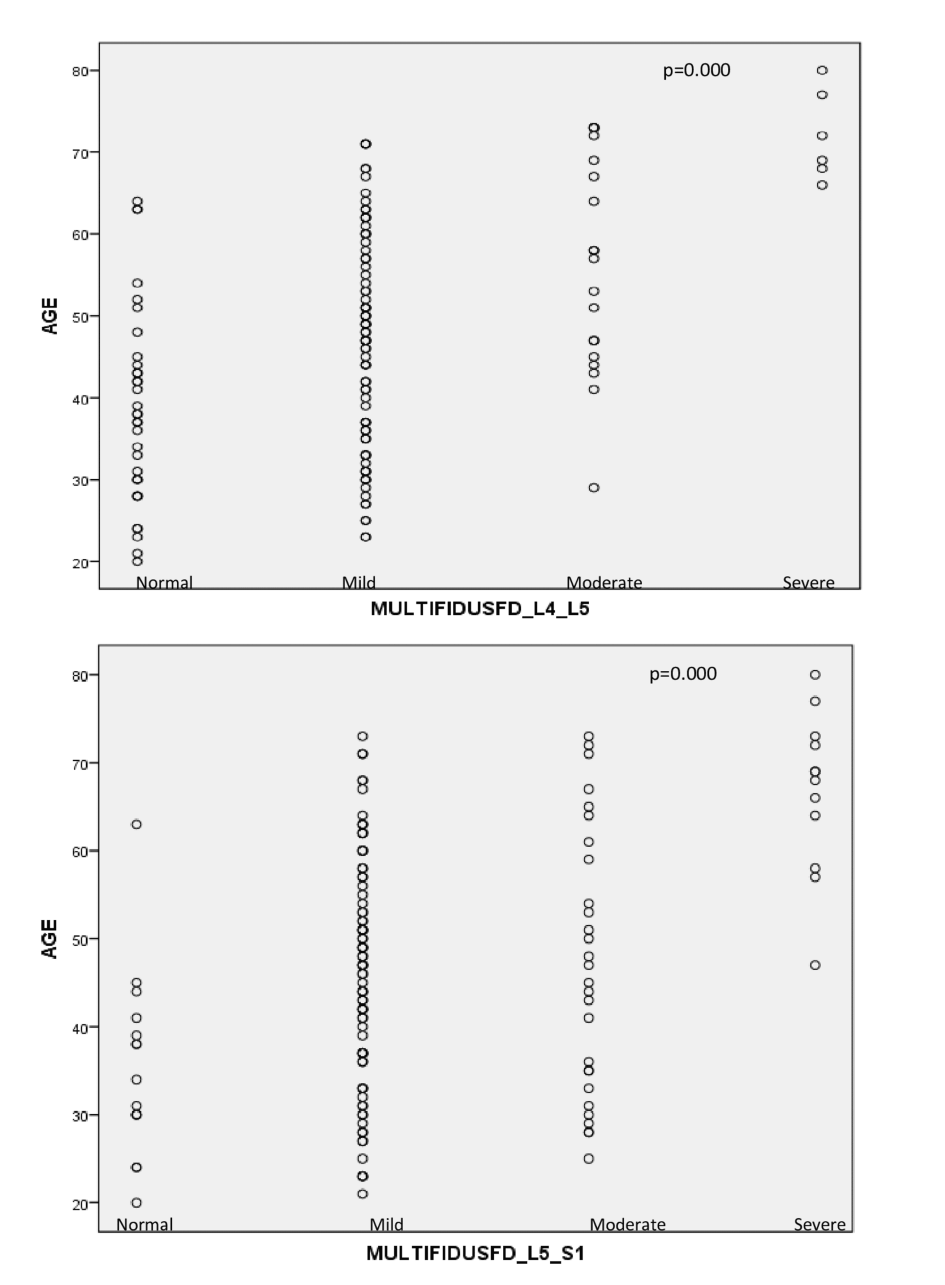
Correlation analysis between age and multifidus fatty degeneration (FD) in a scatter plot

No correlation was found between the herniation degree at the L4-L5 and L5-S1 levels and multifidus muscle fatty degeneration (p-values 0.426 and 0.170, respectively) (Table 3, Table 4, and Figure 3).

**Table 3:** Correlations between age, disc herniation, and multifidus fatty degeneration at the L4-L5 level **Correlation is significant at the 0.01 level (2-tailed) with Spearman's Rho Rho: Spearman's rank correlation coefficient, p: p -value

		Age	Disc herniation L4-L5	Multifidus fatty degeneration L4-L5
Age	Rho	1.000	0.117	0.462
	p	-	0.167	0.000**
Disc herniation L4-L5	Rho	0.117	1.000	0.068
	p	0.167	-	0.426
Multifidus fatty degeneration L4-L5	Rho	0.462	0.068	1.000
	p	0.000**	0.426	-

**Table 4:** Correlations between age, disc herniation, and multifidus fatty degeneration at the L5-S1 level **Correlation is significant at the 0.01 level (2-tailed) with Spearman's Rho Rho: Spearman's rank correlation coefficient, p: p -value

		Age	Disc herniation L5-S1	Multifidus fatty degeneration L5-S1
Age	Rho	1.000	-0.030	0.357
	p	-	0.723	0.000**
Disc Herniation L5-S1	Rho	-0.030	1.000	-0.117
	p	0.723	-	0.170
Multifidus fatty degeneration L5-S1	Rho	0.357	-0.117	1.000
	p	0.000**	0.170	-

There was a significant positive correlation between age and fatty degeneration in the LMM at both levels, with more advanced degeneration seen with increasing age (p<000.1, r values 0.462 and 0.357, respectively) (Table 3 and Table 4). Regarding fatty degeneration grades, significantly more patients had a higher grade with increased age (p<0.001) (Table 5).

**Table 5:** Age comparison between patients according to the lumbar multifidus muscle fatty degeneration grades at L4-L5 and L5-S1 levels Data are presented as mean±standard deviation. *One-way ANOVA (Post hoc Hochberg test)

Fatty degeneration grade	L4-L5 level			L5-S1 level		
	n	Age (years)	p	n	Age(years)	p
Normal	34	38.35 ±11.8	<0.001*	15	35.40 ± 10.6	<0.001*
Mild	81	46.59 ± 12.9		85	46.18 ± 12.8	
Moderate + Severe	25	59.84 ± 13.5		40	52.95 ± 16.2	

## Discussion

This study showed that among patients with chronic LBP, LMM fatty degeneration was mostly seen in females and increased with age. However, we did not find any relationship between disc herniation and LMM fatty degeneration grades at L4-L5 and L5-S1 levels different from some other studies in the literature [10,11].

In biomechanical evaluations of LBP, muscular stabilization of the "neutral zone" in the lumbar region is crucial. The multifidus muscle plays a critical role in stabilizing this zone [12], and dysfunction of the LMM is strongly correlated with LBP [13]. Fat infiltration in the LMM leads to muscle dysfunction, which can be exacerbated by factors such as nutritional disorders, inadequate physical activity, and immobilization. Muscle mass naturally declines around age 40, with an approximate 8% decrease per decade [14]. In our study, we found a significant association between LMM fatty degeneration and older age in females. Ekin et al. reported a higher prevalence of LMM atrophy in individuals aged ≥40 years, irrespective of gender [11]. They also observed a higher occurrence of disc herniation in patients with LMM atrophy at both levels compared to those without atrophy. In contrast to this observation, the present study found no correlation between herniation and muscle fatty degeneration. Related to this result, it could be considered that muscle atrophy is caused mainly by other factors such as immobility, decreased physical activity, and older age.

Numerous histological and imaging studies in the literature suggest that fat infiltration is associated with LBP, indicating a possible link between pain and histomorphological changes in muscles [15-17]. Kjaer et al. proposed that LBP may lead to impaired neuromuscular function, resulting in histological changes in muscles, including atrophy [3]. Additionally, we hypothesized that chronic pain-related poor posture may contribute to LMM dysfunction and subsequent fatty degeneration in the muscle. Consistent with our hypothesis, a study done by Pezolato et al. showed that sedentary individuals with LBP and sway back posture may have morphologic changes in the lumbar erector spinae and lumbar multifidus muscles as a result of both the presence of pain and their habitual posture [18].

Kader et al. highlighted that the LMM, the largest medial muscle in the lumbar spine's lumbosacral region, exhibits significant atrophy in patients with chronic LBP [4]. Given that the L5-S1 and L4-L5 levels are key sites for lumbar spine mobility, we focused our assessment on these areas concerning back pain, herniation, and muscle degeneration.

Our study also demonstrated that the degree of LMM fatty degeneration is higher with increased age and more severe in females. A higher fatty muscle degeneration in females might be related to different biomechanical characteristics between genders. Physical inactivity might be a risk factor for developing fatty degeneration related to increased age.

This study has some limitations, including a relatively small sample size and limited representation of older age groups, which restricts the generalizability of our findings to general populations. Additionally, factors such as BMI-matched groups and degrees of physical activity level were not analyzed in our study. Therefore, larger-scale studies are necessary to provide more definitive insights.

## Conclusions

This study suggests that the prevalence and severity of multifidus muscle fatty degeneration increase with age, especially in females, and show no correlation with lumbar disc degeneration. Considering its effect on chronic LBP, it is often overlooked if it is not examined on MRI. In the future, increasing awareness of multifidus muscle dysfunction and starting early exercise programs related to LMM strengthening may prevent chronic LBP.
